# Data and method for assessing the sustainability of electricity generation sectors in the south Asia growth quadrangle

**DOI:** 10.1016/j.dib.2019.104808

**Published:** 2019-11-16

**Authors:** Imran Khan

**Affiliations:** Department of Electrical and Electronic Engineering, Jashore University of Science and Technology, Jashore-7408, Bangladesh

**Keywords:** Sustainability, Electricity generation, Sustainable development, Sustainability index, South Asia, Assessment methods

## Abstract

The research article “Khan I, Sustainability challenges for the south Asia growth quadrangle: A regional electricity generation sustainability assessment, Journal of Cleaner Production. 243 (2020), 118639, 1–13. DOI: https://doi.org/10.1016/j.jclepro.2019.118639” [1] is linked to this data article. The electricity generation related data were collected from the electricity authorities of Bangladesh, Bhutan, India, and Nepal annual reports, which were publicly available through their websites. Two methods of sustainability assessment, the ‘global’ and ‘multi-criteria decision analysis (MCDA)’ were employed. These two methods were adopted from recent literature. Related data were thus also collected from previous studies in the literature. These two models were explicitly used through a step-by-step calculation using the collected data. These data and methods will allow the researchers to replicate the methods readily. The use of this data and method will also enhance applying a similar approach to other related datasets. Overall, this dataset and method of calculation allow the researcher or analyst to avoid a number of issues: (i) it eliminates considering a large volume of electricity generation data from a myriad of sources for the four countries; (ii) this dataset is ready to be used for any further related sustainability assessment, thus reducing the steps by breaking large datasets down in a way that makes the analysis much easier, and (iii) the calculation steps are ready to be used for any other similar dataset.

## Highlights

• Two sustainability assessment methods are presented for the electricity sector.
• Presents related data for sustainability assessments.
• This dataset can be used as a reference for future sustainability assessment.

**Table:** Specifications Table

Subject	Energy (Sustainability)
Specific subject area	Regional sustainability assessment was conducted using two different sustainability assessment methods.
Type of data	Equation Table
How data were acquired	Data were collected from the annual reports of the respective countries' electricity authorities: - Bangladesh power development board (BPDB) - Bhutan electricity authority (BEA) - Central electricity authority (CEA) in India - Nepal electricity authority (NEA)
Data format	Raw Analyzed Filtered Processed
Parameters for data collection	For the comparison purposes of the results obtained from the global and multi-criteria decision analysis models, global data for the indicators were collected from the literature. Electricity generation related data were collected from 2014 to 2016. The literature survey for data collection was conducted by categorizing the indicators as economic, environmental, and social for the MCDA model. The countries or regions for which total generated energy was not found, installed capacity data was collected.
Description of data collection	For the global model, data were used from [2,3]. For the multi-criteria decision analysis model, indicators' data were collected from the literature and presented as tables in excel file and in [1]. Energy generation and capacity related data were collected from each country's electricity authority's website.
Data source location	Institution: - Bangladesh Power Development Board (BPDB) - Bhutan Electricity Authority (BEA) - Central Electricity Authority (CEA) in India - Nepal Electricity Authority (NEA) Region: South Asia Growth Quadrangle (SAGQ) Countries: Bangladesh, Bhutan, India, and Nepal
Data accessibility	With the article
Related research article	Author's name: Imran Khan Title: *Sustainability challenges for the south Asia growth quadrangle: A regional electricity generation sustainability assessment* Journal: *Journal of Cleaner Production* DOI: https://doi.org/10.1016/j.jclepro.2019.118639

**Table:** 

**Value of the Data** • These data can be used to assess the global sustainability of any electricity generation system. • Sustainability policymakers, researchers, engineers, and academics can use the data along with the detailed step-by-step calculation to obtain the global sustainability of any electricity generation systems. • The data are presented along with the detailed calculation steps for the global model, thus changing the related values at the respective field will generate new results for the system under consideration. • These detailed calculation steps and data for the global sustainability assessment model will reduce the time-consuming calculation steps for any researcher who intends to use this model. • For the multi-criteria decision analysis model, data are collected from the literature. This will reduce the time for other researchers who are in search of sustainability assessment-related indicators' data in the literature. • Sustainability assessment is a dynamic process and needs to be evaluated at certain time intervals to check its status. Therefore, this dataset can be used as a reference for any future electricity generation related sustainability assessment for the south Asia growth quadrangle

## 1 Data

All the data are stored in one excel file containing a number of sheets (see supplementary data in the Appendix A). The first sheet ‘*Equations*’ shows the equations used for the sustainability assessment for the global model and MCDA model. The next ten sheets namely *SI-Coal, SI-Oil, SI-Gas, SI-Small Hydro, SI-Large Hydro, SI-Solar, SI-Wind, SI-Biomass, SI-Geothermal,* and *SI-Nuclear* show step-wise detail calculation associated with data for the global model for sustainability assessment. The sheet ‘*Global SI*’ presents the calculated sustainability index for the different electricity generation technologies. The next five sheets *Bangladesh, India, Bhutan, Nepal,* and *SAGQ* used the calculated sustainability index from the global model and applied it to either total generated electricity or total installed capacity for the years 2014, 2015, and 2016. The sheet ‘*Country-SAGQ SI*’ shows country-specific and regional (i.e., SAGQ) sustainability index obtained from the global model. The last two sheets ‘*Eco & Env*’ and ‘*Social*’ present the data used for the MCDA model to assess sustainability. These are listed in Table 1.

**Table 1:** Data and method.

Sustainability Models		Main Equations Used	Calculations/Method	References/Data Sources
Global Model		$V_{i}=\frac{1-\text{exp}\left(-m_{i}*{\left(\frac{\left|P_{i,\;T\;}-P_{i,min}\right|}{n_{i}}\right)}^{A_{i}}\right)}{1-\text{exp}\left(-m_{i}*{\left(\frac{\left|P_{i,\;max\;}-P_{i,min}\right|}{n_{i}}\right)}^{A_{i}}\right)}$ (1) $SI_{T}=\overset{i=16}{\underset{i=1}{\sum }}\alpha_{i}*\beta_{i}*\gamma_{i}*V_{i}$ (2)	In the excel file, sheets: *SI-Coal, SI-Oil, SI-Gas, SI-Small Hydro, SI-Large Hydro, SI-Solar, SI-Wind, SI-Biomass, SI-Geothermal, SI-Nuclear, and Global SI*	[2,3]
		$SI_{System}=\overset{T=T_{Tot}}{\underset{T=1}{\sum }}\frac{E_{T}}{E_{Tot}}*SI_{T}$ (3) $SI_{System}=\overset{T=T_{Tot}}{\underset{T=1}{\sum }}\frac{C_{T}}{C_{Tot}}*SI_{T}$ (4)	In the excel file, sheets: *Bangladesh, India, Bhutan, Nepal, and SAGQ*	[3]
MCDA Model		For weight assignment: if N is the number of total technologies considered, then the weight (*w*) of the indicator for kth technology will be: $w_{k}=\left(\frac{1}{N}\right)\overset{N}{\underset{i=k}{\sum }}\left(\frac{1}{i}\right)$ (5) Here, $w_{1}\geq w_{2}\geq \;\;.\;.\;.\;.\;.\;.\;.\;.\;\geq w_{k}$, $w_{1}+w_{2}+\;.\;.\;.\;.\;.\;.\;.\;+w_{k}=1,$ N = 10, and k = 1 to 10.	Detailed method was adopted from [4]. For this, data were used from sheets: ‘*Eco & Env’* and ‘*Social’*.	[4,5]
		$W_{T}^{c}=\underset{indicators}{\sum }w_{k}$ (6) Where: $W_{T}^{c}$ : Total score reflecting the performance of technology T on criterion c. T: Generation technology; e.g. coal, gas, oil, hydro. c: Criterion; e.g. economic, environmental, social. $w_{k}$ : Indicator specific assigned weight obtained from equation **(5)**.	Adopted from [4].	[4]
		$SI_{T}=\underset{criteria}{\sum }\left(\frac{1}{N_{c}}\times W_{T}^{c}\right)$ (7) Where: SI_T_: is the technology specific sustainability index. $N_{c}$ : Number of indicators for criterion c. $W_{T}^{c}$ : Total score reflecting the performance of technology T on criterion c.	Adopted from [4].	[4]
		$SI_{System}=\overset{T=N}{\underset{T=1}{\sum }}\frac{C_{T}}{C_{Tot}}\times SI_{T}$ (8) Where: SI_System_: Overall electricity generation system sustainability index. C_T_: Output capacity (MW) of the technology in the future (or present) year. C_Tot_: Total system capacity (MW) in that future (or present) year.	Adopted from [4].	[4]

## 2 Experimental design, materials, and methods

**Fig. 1:**
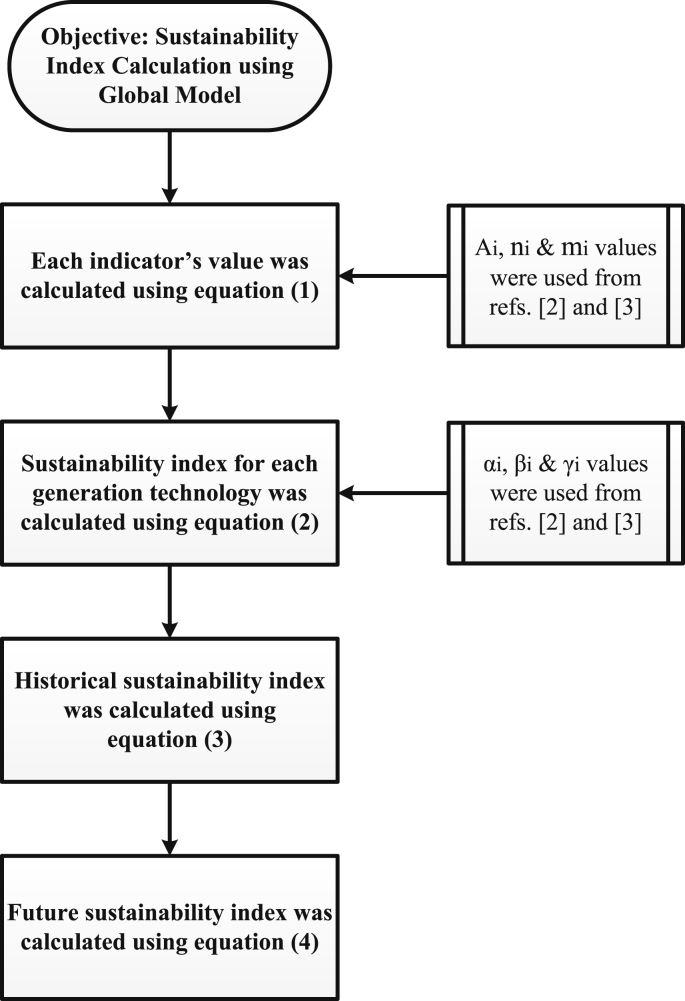
Method of calculation for the Global sustainability index assessment model.

**Fig. 2:**
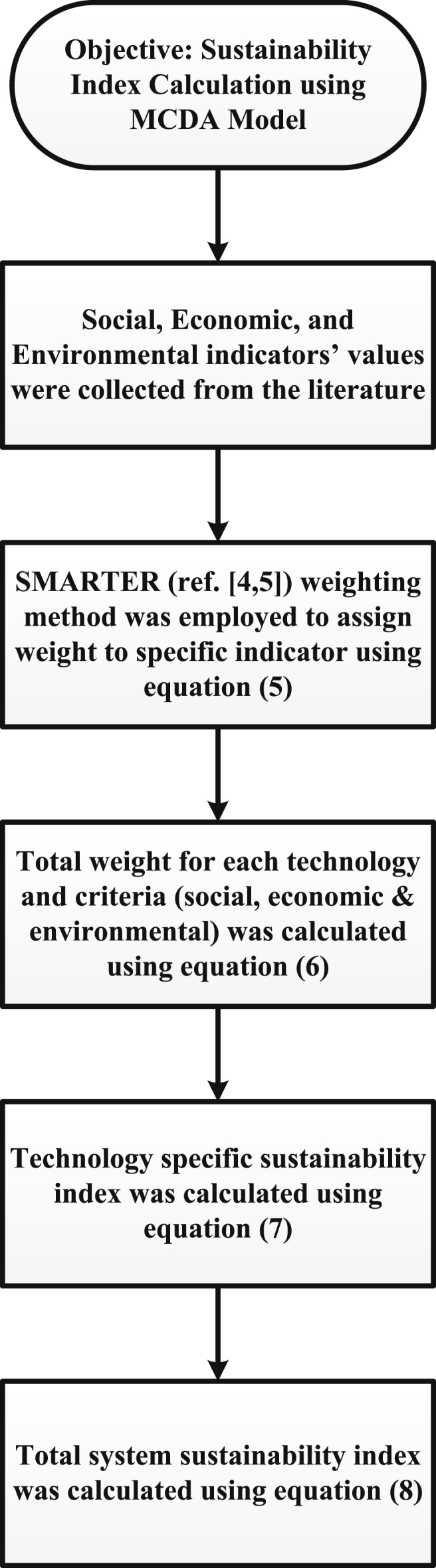
Method of calculation for the MCDA sustainability index assessment model.

The calculation equations and use of data are listed in Table 1. Step by step calculation methods for the Global and MCDA models are illustrated in Fig. 1, Fig. 2, respectively.

The method of calculation for the global sustainability index model comprises four basic steps and is illustrated in Fig. 1. In the first step, different values for different factors (i.e., A_i_, n_i_, m_i_) were used from [2,3] and each indicator in social, economic, and environmental criteria was calculated using equation (1). The sustainability index for each generation technology was calculated in the second step. References [2,3] were also used for the values of α_i_, β_i_, and γ_i_ using equation (2) to obtain the technology-specific sustainability index. The final two steps calculate the historical and future sustainability index using equations (3) and (4), respectively.

On the other hand, there are five steps to calculate the sustainability index of the power generation system through the MCDA model. In the first step, social, economic, and environmental indicators were collected from the literature. The list of articles used can be found in the excel file in the sheets named as- ‘*Eco & Env’* and ‘*Social’*. SMARTER (Simple Multi-Attribute Rating Technique Exploiting Ranks) method was then employed to assign a weight to the indicators using the equation (5) as shown in Table 1. Details of this weighting method can be found in Refs. [4,5]. Using equation (6), total weights for each technology and criteria (i.e., social, economic, and environmental), were calculated. The sustainability index for each technology was then calculated through equation (7). Finally, the system sustainability index was calculated using equation (8). All these steps are depicted in Fig. 2.

## Conflict of interest

The authors declare that they have no known competing financial interests or personal relationships that could have appeared to influence the work reported in this paper.
